# Kinship Trouble: What, When, Where, Why, and How—and So What?

**DOI:** 10.1017/s0959774326100389

**Published:** 2026-04-14

**Authors:** Sabina Cveček, Maanasa Raghavan, Penny Bickle

**Affiliations:** 1 Field Museum of Natural History, Negaunee Integrative Research Center, 1400 S Lake Shore Dr, Chicago, IL 60605, USA; 2 Austrian Academy of Sciences, Austrian Archaeological Institute, Dominikanerbastei 16, 1010 Vienna, Austria; 3 Department of Anthropology, University of Illinois at Chicago, 1007 West Harrison St, 2102 BSB Chicago, IL 60607, USA; 4 American Museum of Natural History, Division of Anthropology, 200 Central Park W, New York, NY 10024, USA; 5 Department of Human Genetics, Cummings Life Science Center, University of Chicago, 920 E 58th St, Chicago, IL 60637, USA; 6 Committee on Genetics, Gemomics, and System Biology, Cummings Life Science Center, University of Chicago, 920 E 58th Street, Chicago, IL 60637, USA; 7 Department of Archaeology, University of York, The King’s Manor, Exhibition Square, York YO1 7EP, UK

## Abstract

*What* is kinship trouble? *When* and *where* did it emerge? *Why* does it matter and *how* can we overcome it? These questions guide our discussion of kinship trouble, a term meant to capture the difficulties in reconstructing ancient kin relations, but also an attempt to resolve them through interdisciplinary collaboration and ethically adequate approaches. Motivated by the importance of crossing disciplinary boundaries and the urgency of working together to understand human diversity in the past and present, we reconsider kinship not only as a biological or genetic but also as a social phenomenon for the study of societies through archaeogenetic, archaeological, and socio-cultural anthropological approaches. As to the question of *how* kinship trouble could be overcome, we propose making more ‘oddkin’ (*sensu* Haraway) to bring disciplines into the conversation and foster unexpected collaborations around three themes: ethical collaboration, the integration of biological and social approaches, and kinship studies as acts of care and (non)mutuality of being.

## Introduction

This paper introduces a special thematic issue on *Kinship Trouble: Traversing Interdisciplinary Boundaries between Archaeology, Archaeogenetics and Socio-cultural Anthropology* by reviewing the state of the field, structured around five key questions: the *what*, *when*, *where*, *why*, *how* of ‘kinship trouble’. We define ‘kinship trouble’ as a term that captures difficulties in reconstructing ancient kin relations, but also as an attempt to resolve them through interdisciplinary collaboration and ethical approaches that engage all contributing disciplines as well as various stakeholders (academic and non-academic institutions, museums and communities, as relevant) throughout the research process.

First, it defines *what* kinship trouble is by asking what it is that archaeology, archaeogenetics and socio-cultural anthropology want to understand about kinship and its role within global history. Second, the paper follows the historical contribution of these three subfields of interest to the special edition towards kinship studies to ask *when* and *how* kinship trouble emerged. Third, by critically discussing empirical examples of kinship trouble, the discourse will be rooted in time and place, addressing *where* kinship trouble appears. Next, the paper addresses *why* kinship trouble matters. We argue here for the critical urgency of understanding kinship again^1^ not only as a biological but also as a social phenomenon for the study of societies through archaeogenetic, archaeological and anthropological approaches, and hence the call for multi-disciplinary approaches. The timeliness of this discussion is underscored by ever-increasing investigations on kinship and biological relationships within these fields that currently, for the most part, take on either singular perspectives or partial input from the other fields. The paper concludes with suggestions and perspectives on *how* kinship trouble could be disentangled in an attempt to bring the three disciplines into conversations with each other around three themes: ethical collaboration; the integration of biological and social approaches; and kinship studies as acts of care.

Several contributors to this special edition have presented preliminary versions of their papers at the Archaeology Division-sponsored session organized at the 2023 American Association of Anthropologists Annual Meeting in Toronto, organized by Sabina Cveček, and a follow-up workshop at the 2024 American Institute of Archaeology Annual Meeting in Chicago, organized by Sabina Cveček, Maanasa Raghavan and Penny Bickle. The meeting in Chicago bears historical importance, as it was held 40 years after David M. Schneider (1984), at the time a professor at the University of Chicago, called to abandon kinship studies in anthropology.

This special thematic edition critically considers the contributions of the three relevant fields to the understanding of kinship in the deep past, as well as issues that have particularly emerged from the implementation of new methods, such as biomolecular analyses, to reconstructing ancient kinship patterns. The proposed collection of papers combines innovative methods with theoretical approaches that push existing boundaries in the field that are of interest to a wide readership. It attempts to be global in scope, and it unpacks the issue of kinship and belonging in prehistory as well as in the present. This special thematic issue also seeks to bridge the gap between symbolic and materialist approaches to kinship as well as the European and American schools of archaeology and/or anthropology. As kinship still needs anthropologists in the twenty-first century (Cveček 2024), at the heart of this volume stands addressing the topic of human kinship in ancient societies in interdisciplinary and ethical ways.

Currently, archaeogenetics research is more likely to involve collaborations between archaeologists and geneticists, while socio-cultural anthropologists are still rarely included in these discussions. The willingness to cross boundaries between archaeology, anthropology and archaeogenetics is, in this special issue, reflected through the integration of scholars from these disciplines. A wide variety of regional specialists will speak to the broader archaeological, anthropological and archaeogenetic communities. Bringing together expert scholars on the topic, this special issue will be of interest to scholars who approach kinship through biological approaches, those who understand kinship as an exclusively social construct, as well as those who consider it to be a mixture of the two. It seeks to engage not only specialists and students in archaeology, socio-cultural anthropology, and biological anthropology but also an interdisciplinary audience of social scientists, including scholars in cultural studies, science and technology studies, and history.

## *What* is kinship trouble?

We begin our inquiries into ancient kinship by treating kinship not as given and easily reconstructed through a single method or discipline, but as a trouble that requires new empirical and theoretical inquiries. While socio-cultural anthropology has a rich history of research into the topic of kinship spanning a century and a half, archaeology has largely avoided the topic of kinship since the 1960s. Instead, with the explosion of archaeogenetic investigations in the last decade, archaeologists found new venues for addressing biological relatedness between ancient and recent individuals. The use of ancient DNA (aDNA) to reconstruct biological relationships has uncovered unique insights into past societal structures by using genetic relatedness as a proxy for the degree of kinship (Vai *et al*. 2020). However, this poses a ‘trouble’ since kinship is much more than a reconstructed genetic pedigree between individuals buried together. In practice, kinship may or may not overlap with genetic proximity. Hence, ancient DNA methods in and of themselves pose a challenge to translating genetic ties into kinship terms and practices originally grounded in anthropological inquiry that encompass many more facets of individual and group identities than biological and/or genetic ties alone.

If we consider kinship as ‘those biological and/or social ties arising from the union of persons … and which determine the belonging and social identity of the children born to or adopted by this couple’ (Godelier 2011, 566), important kinship ties can be created through adoption (Goody 1969; Howell 2009; Terrell & Modell 1994), fostering (Alber 2013; Leinaweaver 2014), child circulation (Alber 2013; Leinaweaver 2008; 2014), affinal kin ties through marriage (Brettel 2017; Fox 1983; Lévi-Strauss 1949; 1969; Mody 2015; Radcliffe-Brown & Forde 1950), milk kinship (El Guindi 2020; Parkes 2001) and/or co-residence (Carsten 1997; 2000; Lévi-Strauss 1982) among other kinning practices that cannot be easily captured through ancient DNA datasets. These ‘fictive kin’^2^ relationships functioned to integrate adults into informal supportive networks that surpassed formal kin obligations conventionally prescribed by blood or marriage (see **Herrero-Corral**, this issue). Moreover, the practice of informal adoption of enslaved children speaks to the salience of relatedness and co-residence (Guttman 1976, 226–8), further highlighting the diversity and complexity of kinning practices.

Kinship, however, may not be limited to humans. Considered as ‘mutuality of being’ (Sahlins 2013), kinship may extend to humans, but also animals and plants and interconnect the living and the non-living since they all ‘participate intrinsically in each other’s existence’ (Sahlins 2013, ix). Again, many of those (non)human relations escape current ancient DNA analysis. However, a recent attempt has been made toward considering the mutuality of being between the human and non-humans in genomics coming from Aotearoa, recognizing Te ao Māori, the local worldview of interconnectedness of the living and the non-living (Te Aika *et al.* 2025). Moreover, kinship is not only about the mutuality of being (cf. Sahlins 2013), but also concerns the non-mutuality of it (Goldfarb & Bamford 2024; Goldfarb & Schuster 2016), when kin ties are (in)voluntarily broken. Kinship as non-mutuality of being does not manifest in solidarity and care but also in exclusion, disconnection, neglect and violence (Carsten 2019; Franklin & McKinnon 2002; Goldfarb & Bamford 2024; Goldfarb & Shuster 2016). Likewise, kinship rules not only include but also exclude biologically closely related individuals, such as siblings of different genders in a unilineal descent system (Cveček & Gingrich, this issue; Evans-Pritchard 1940). All of these aspects pose a challenge for grasping kinship in its full array through genetic methods alone, since inferred genetic pedigrees from archaeological contexts may assign a genetic kin relation in cases when those relations may have been broken or not infer a relation where there may have been strong kin relations. Since many of the perceived and practiced kinship ties are effectively ‘hidden’ from the genetic record by consequently the inferred genetic pedigrees could be erroneous on the level of relevant social practice.

We are not the first to address the mismatch between the biological and social aspects of kin concerning genetics. In response to the rise of sociobiology (Wilson 1975), Marshall Sahlins in *The Use and Abuse of Biology* (1976) argued that kinship ‘is not [a] genetic connection but quite generally … people of the same “kind”; a notion of social identity’ (Sahlins 1976, 26). For this reason, ‘the categories of “near” and “distant” [kin] vary independently of consangunial distance’ (Sahlins 1976, 112). Five decades later, this remains a timely insight. Tim Ingold (1991, 361, italics in original) also pointed out that

genetics has to do with (quantitative) coefficients of related*ness*, not with the (qualitative) dynamics of relation*ship*, and however each might be linked as cause, conditions or consequence of the other, they are not the same. Genetic kinship, then, is kinship cut out from the context of relationships and ascribed as the common property of individuals regarded as discrete, isolated, and countable entities.

Therefore, instead of letting genetic kinship, or genetic relatedness as is proxied by genetic data, be cut out of wider everyday, socio-political relations and contexts, an important next step in kinship analysis in the past is to acknowledge a multidimensional framework in which close genetic relatedness is seen as possibly overlapping with—but not necessarily equal to—kinship. In fact, some well-contextualized archaeological and archaeogenetic analyses have distinguished between kinship and genetic pedigree (see Cummings & Fowler 2023; Fowler *et al*. 2022; Rivollat *et al*. 2023; Villalba-Mouco *et al*. 2022; Yüncü *et al*. 2025). However, more often than not, scholars continue to translate more linear concepts of genetic relatedness into kinship structures, such as genetic relatedness via paternal line as the practice of patrilineal descent (Fowler *et al*. 2022; Rivollat *et al*. 2023) and genetic relatedness via maternal line as the representation of female lineages (Yüncü *et al*. 2025). Such interpretations obfuscate the fact that descent and lineage are largely social and imaginary rather than genetic phenomena (see below).

By treating genetic relatedness as a distinct layer of inferred human relationships, we may use it to question whether and if genetic relations mattered at death and/or in performative burial contexts while being aware that the inferred genetic pedigrees may not equal kinship. This becomes critical as kinship concepts and terms, such as ‘female exogamy’ (see Cveček & Gingrich, this issue), are often borrowed from socio-cultural anthropology but may, in practice, be attributed to a much narrower definition that is limited by the scope and nature of genetic data. Without appropriate discussion or consideration of alternative models, this can lead to misinterpretations and disconnect between the fields. This led us to coin a term—kinship trouble—that encompasses the difficulties in reconstructing ancient kin relations, but also an approach that could be resolved through interdisciplinary collaboration and ethical approaches.

## Kinship trouble

**Figure 1.**
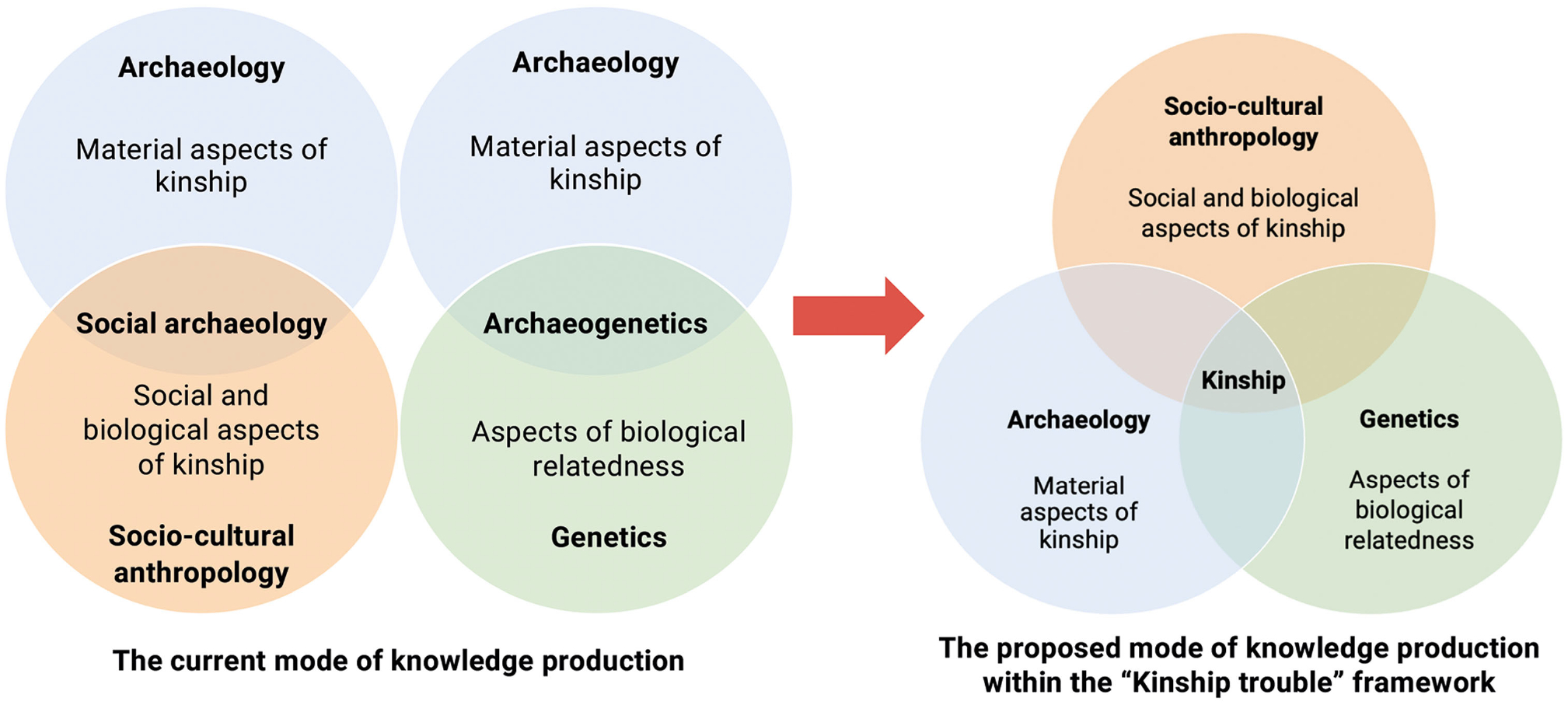
The current mode of knowledge production and a proposed mode of knowledge production within the ‘Kinship trouble’ framework.

‘Kinship trouble’ as the overarching notion for this special edition is inspired by Judith Butler’s *Gender Trouble* (Butler 1990), which challenged the division between sex and gender to show that both are performative and created, maintained and perpetuated through interaction with each other. The term is also proposed by Butler in the UCL Housman lecture^3^ titled *Kinship Trouble in the Bacchae*, given in 2017. Here, we propose ‘kinship trouble’ has two meanings pertinent to the current disciplinary debate. First, ‘kinship trouble’ closely resembles ‘gender trouble’ in that it challenges the division between kinship as biological and kinship as a symbolic and cultural construct. Kinship, as defined in contemporary anthropological understanding, ‘has to do with cultural systems that include biological facts, not biological systems that include cultural facts’ (Read 2018, 15). With so much ancient DNA analysis now undertaken on archaeological collections, it is timely to promote an improved understanding of kinship as a set of performative acts and relations that might, but need not necessarily include, a biological component. Kinship, like gender, is about *becoming*^4^ rather than being and this is where inferred genetic relations between individuals, currently reduced to the first-, second- or tenth-degree relatives, fall short. Second, ‘kinship trouble’ historically reflects an intellectual challenge, if not trouble, with addressing kinship in archaeology, socio-cultural anthropology and bioanthropology. By giving voice to experts in these disciplines, the collection of papers presented here offers a balanced, interdisciplinary and synoptic overview of cutting-edge research into one of the most important questions in archaeology today and proposes a new mode of knowledge production within the ‘kinship trouble’ framework in Figure 1. Therefore, we hope that this special thematic issue will be of broad interest to a diverse, global scholarly audience, including archaeologists, socio-cultural anthropologists and archaeogeneticists.

## *When* and *where* has kinship trouble emerged?

The following section traces how anthropological theories of kinship—from Morgan’s nineteenth-century evolutionism, through British functionalism and French structuralism, to feminist critiques, the concept of house society and new approaches to kinship—have shaped archaeological approaches to social organization. It highlights how descent, alliance, residence, gender, substance and personhood have been variably emphasized, critiqued and reconfigured, creating both enduring concepts and significant limits that remain central to archaeogenetic debates today. Building on this intellectual history, the following sections examine how aDNA approaches have reintroduced kinship to archaeology, why this resulted in ‘kinship trouble’ and how interdisciplinary collaboration can address its conceptual, methodological and ethical challenges.

## Early evolutionist theory of kinship: From classificatory to descriptive kinship systems

Anthropologists began systematically studying kinship in the nineteenth century, most famously through Lewis Henry Morgan’s 1871 work *Systems of Consanguinity and Affinity of the Human Family*. Morgan focused on kinship terminology—now commonly grouped into six or seven universal types (following, in part, Murdock 1949). Morgan aimed not only to document the global diversity of kinship systems but also to explain it. He did so through the lens of nineteenth-century social evolutionism, which assumed that societies progressed over time from ‘primitive’ to ‘advanced’ forms. Morgan drew a distinction between different kinship systems based on kin terms. For example, in classificatory systems, such as the Iroquois, the same term is used for both ‘father’ and ‘father’s brother’, while ‘mother’s brother’ has a distinct term. In contrast, the English descriptive system assigns a different term to each of these relatives. In this framework, the ‘classificatory’ system he described among the Iroquois, who reckoned their ancestors via matrilineal descent, was seen as an early stage of that evolutionary sequence. Morgan argued that over time, classificatory systems developed into ‘descriptive’ systems found in languages like English. In this case, people remember ancestors from both mother’s and father’s sides of the family, a practice known as bilateral descent, supposedly marking ‘civilization’.

Soon after the volume was published, it was critiqued, notably by Franz Boas (1908), for its social evolutionary underpinnings and the lack of ethnographic data at the time to make such broad, evolutionary claims. However, this early debate set the systematic inquiry into kinship systems as one of the central tasks for anthropologists during the following decades. These two early works, we argue here, created two strands in archaeological thought that in some ways still persist in approaches today. First, kinship was seen as culturally varied, and changing through time. Perhaps this first strand also guided the archaeology that followed in the first half of the twentieth century, by regarding kinship as more relevant to other topics. For example, Gordon Childe (1950) claimed metal-working provided the beginnings of ‘emancipation from kinship’ during the Bronze Age, implying a preceding level of a society organized by kinship as less complex, but also suggesting that to archaeologists of later periods kinship would not be a significant form of social analysis (Cveček 2023). Second, Morgan’s focus on terminology, kin labels and spoken language as a crucial route to understanding the overall organization of kinship provided the sense that kinship systems were much less accessible through the material record. This, however, has not stopped archaeologists such as Andrew Sherratt (1981) and Marija Gimbutas (1991) from proposing, in a manner inspired by Morgan’s evolutionary theory, that Neolithic societies in Old Europe were matrilineal, whereas Bronze Age societies were patrilineal (see Cveček 2025a). Although, throughout this time, methods of analysis were debated, the central role of kinship to both anthropology and our understanding of past societies was not challenged.

## British functionalism and French structuralism: descent and alliance as the core of kinship

With the rise of functionalism in the United Kingdom, the study of kinship and descent became a key topic in socio-cultural anthropology during the first half of the twentieth century. The British functionalist school critically advanced the study of kinship by focusing on descent as a binding principle in non-state societies (Evans-Pritchard 1940; Fortes & Evans-Pritchard 1940; Radcliffe-Brown 1941). Many of those kinship concepts, including descent and residence rules, remain relevant for the study of kinship in socio-cultural anthropology, archaeology and archaeogenetics today. As they are currently widely used in archaeogenetic publications, we provide an overview of some of these concepts below.

Today, socio-cultural anthropologists understand descent as a practice when ties of filiation (links between parent and child) ‘are repeated generation after generation, and if the social emphasis is on the whole series of such links, backwards into preceding generations and, prospectively, forwards into future ones’ (Parkin [1997] 2003, 15). Descent is not a biological principle but an imagined chain of links that connect a person with past and future generations. Descent may, but not necessarily must, coincide with the biological proximity between a person and their predecessors and/or successors. Descent also differs from lineage, which is ‘a group descended through the male or the female line from one of the sons or daughters of a clan founder or one of their known descendants … members hold material and immaterial assets in common, help each other and show solidarity with each other … A lineage acts as a corporate group with respect to other lineages’ (Godelier 2011, 567). Like descent, lineage is also not a biological principle but an imagined chain of links between a person, their ancestors and future generations.

The *descent rule* is a principle or a criterion that defines ‘a person’s belonging, at birth, to a group of persons claiming to descend from one or several common ancestors’ (Godelier 2011, 560). Nevertheless, birth is not the only way to become part of a descent or lineage group. A person can become a member of a descent group through adoption and milk parenthood (El Guindi 2020), which shows that biological proximity plays a marginal role in the tracing of descent. Descent can manifest in many different ways, including patrilineal or agnatic descent, matrilineal or uterine descent, ambilineal or duolineal descent, and bilineal descent, which can be further divided into cross-bilineal descent and parallel-bilineal descent, as well as bilateral descent (Parkin [1997] 2003). Societies with the same descent rules, however, can display considerable variation in other socio-political traits, such as (de)centralized organization or gender norms (see Cveček 2025a).

Apart from descent rules, the British functionalist school also differentiated between limited options of cross-cultural postmarital residence rules. Postmarital residence patterns are shared rules not set in stone throughout the same tribal group. Postmarital residence may vary throughout the lifecycle or throughout society and therefore should be perceived as a matter of tendencies and preferences rather than hard rules (Parkin [1997] 2003, 28–32).^5^ Types of postmarital residence rules include patrilocal or virilocal residence, matrilocal or uxorilocal residence, ambilocal or biological residence, neolocal residence, duolocal residence, avunculocal residence and amitalocal residence (for definitions, see Parkin [1997] 2003, 31). Similar to descent rules, societies that share the same residence rules may nevertheless exhibit substantial variation in other socio-political characteristics, including the degree of (de)centralization and prevailing gender norms (see Cveček 2025a). Until recently, apart from a few exceptions (Ensor 2013; Gimbutas 1991; Sherratt 1981; Souvatzi 2017), archaeologists would not tend to identify modes of descent or residence from archaeological material alone. However, with the rise of ancient DNA and isotopic studies—that allow for identifying mobility patterns in the past—descent and residence rules are back on the wider archaeological agenda. The same applies to identifying marriage alliances, based on the inference of reproductive partners through archaeogenetic methods. Marriage alliances, however, were an important topic of a wave of kinship studies that emerged in response to British functionalism in the late 1940s in France. First published in French in 1949 and then issued in English in 1967, *The Elementary Structures of Kinship* (Lévi-Strauss 1949; 1969), built upon the insights that most clans and lineages are exogamous.^6^ Claude Lévi-Strauss (1969) developed an argument that the incest taboo is a cultural universal rather than a biological feature in all human societies. Employing comparative analysis of rules of kinship and marriage (or comparative ethnology), he developed an important theoretical model based on a cross-cultural comparison of the exchange of women at marriage. Instead of descent or filiation, Lévi-Strauss’s (1969) alliance theory argued that repetition of the same marriage practices over generations defines kinship of social groups and importantly advanced the understanding of kinship not as a biological phenomenon but as a cultural principle. By prioritizing affinal ties^7^ in his analyses, he was able to show that culture (alliance) rather than nature (biology, genes) binds social groups together into a social whole.

## Kinship, gender, and house: a critique of prioritizing descent or alliance for becoming kin

Following the Boasian tradition in the US academic context, forty years ago, David Schneider published *A Critique of the Study of Kinship* (1984), a book that called for the abandonment of kinship studies in anthropology. Schneider claimed that kinship study was a product of Western bias and challenged its use as the universal measure of the study of social structure. At the same time, a feminist critique of kinship emerged following the Wenner-Gre-funded conference titled *Feminism and Kinship Theory*, in which the co-editors and students of Schneider advocated, much more constructively than their teacher, for gender and kinship to be understood and studied as a single topic of study (Collier & Yanagisako 1987). They called for anthropologists to explain why kinship or gender is constituted the way it is in any particular society, rather than *a priori* assume that descent and alliance play important roles.

A third critical development around this time, that crucially contradicted but also complemented both the initial Schneiderian critique of kinship studies and the feminist perspectives on kinship, was Lévi-Strauss’s (1982) concept of *société à maison* or ‘house society’. With the latter concept, Lévi-Strauss (1982) solved the long-standing enigma of Kwakwa̱ka̱’wakw social organization, a tribal group located on the islands and the mainland northwest of Vancouver. He proposed that houses among the Kwakwa̱ka̱’wakw cross-cut incompatible categories (or ‘logical oppositions’ as Lévi-Strauss understood them) such as patrilineal *versus* matrilineal, patrilocal *versus* matrilocal, endogamous *versus* exogamous categories of descent, postmarital residence and marriage patterns. Lévi-Strauss (1982) argued that ‘house societies’ were universal; a model to stand alongside concepts such as forms of descent, and which had also existed in medieval Europe, Japan in the Heian period, and ancient Greece. These critiques and new developments in kinship studies in socio-cultural anthropology have resulted in a profound transformation of kinship studies in socio-cultural anthropology.

## Gender, substance, and personhood: Relatedness and studying emic ways of becoming kin

Kinship remains an important topic of undergraduate and graduate training in the European Union, Canada, the United Kingdom, Australia, New Zealand and most parts of southeast Asia, as well as at some departments in the United States. Hence, Schneider’s critique has not resulted in any global abandonment but in the transformation of kinship studies in anthropology. For example, Janet Carsten, unlike Schneider (1984), proposed not to abandon kinship but to study it in new ways and specifically by focusing on three central concepts—gender, substance and personhood—and trying to understand their emic^8^ perceptions of *becoming* kin. This can help us move beyond genealogical and biological reasoning of kinship (Carsten 1997; 2000; 2004) via descent or alliance alone, which was the driving force of evolutionist, functionalist, and structuralist ideas of kinship (see above). The so-called ‘new kinship studies’^9^ in socio-cultural anthropology work against two important binary oppositions. First, they reject the structural-functionalist^10^ models of kinship in tribal societies *versus* those of the West. Instead, proponents seek to explore emic perspectives on relatedness in each society, encompassing both biological and non-biological kinship ties and the many ways of making kin via relatedness.

In *Cultures of Relatedness*, Janet Carsten (2000) proposed two ways of approaching relatedness: (1) to explore indigenous idioms of being related; and (2) to move away from analytic opposition between biological and social aspects of kinship. This concept of relatedness allows for a more nuanced and open-ended exploration of the diverse ways that humans form and experience kinship ties. Alongside these socio-cultural anthropological critiques, other areas of the discipline have continued an interest in kinship, such as human behavioural ecology (Hrdy 2009) and linguistics (Ball 2018), which **Mittnik and Bentley** (this issue) argue should be integrated with archaeogenetic and material correlates to reveal past kinship systems. At the same time, socio-cultural anthropology today offers a wide array of ‘old kinship studies’ referring to ethnographic studies before Schneider’s intervention and ‘new kinship studies’ that take ways of *becoming* kin at the heart of their analyses. Both approaches can be integrated into the study of past kinship systems to evaluate theories of kinship and social change over millennia, with archaeology providing the primary source of evidence.

## Beyond kinship and back again? House societies and ancient DNA in archaeology

In the early 2000s, following pioneering work by Joyce and Gillespie (2000) among others, archaeology became aware of the earlier major shifts in socio-cultural anthropology. The anthropological concept of ‘house societies’ particularly was extensively adopted in the interpretation of socio-political organization in diverse archaeological contexts (Gillespie 2007; Joyce & Gillespie 2000) and as a more convenient model for addressing property transmission and social memory (see Hodder & Cessford 2004). Following the household archaeology of the 1980s and beyond (Souvatzi 2008; Tringham 1991; 2015; Wilk & Rathje 1982), ‘which was created to move away from deterministic kinship studies’ (Wilk, pers. comm. 2017), archaeologists aimed to address socio-political and economic relations between and within households. However, the house society model is not ‘free’ of kinship ties, such as descent or alliance. Instead, the house society, as Lévi-Strauss (1982) envisioned it, refers to groups who do not recall descent unilineally, namely in a patrilineal or matrilineal manner, but follow both lines of descent, via mother’s and father’s side, bilaterally. **Whiteley** (this issue) elaborates on why and how kinship should be brought back into the house rather than the ‘house society’ model replacing the notion of kinship in both ethnographic and archaeological contexts.

Moving forward to the recent decades, there has now been what **Frieman and Schuster** (this issue) call a ‘palaeogenomic tsunami’ of research in this field since the application of next-generation sequencing technologies for aDNA studies in the late 2000s. This work often bypasses previous discussions about kinship in socio-cultural anthropology and household archaeology, instead drawing directly on terms uncritically and/or erroneously borrowed from anthropological models of kinship to speak about the biological relationships thus recovered (see Cveček 2024). For example, both a recent article in *Science*, arguing for kin-based social inequality in Bronze Age Europe (Mittnik *et al*. 2019), and an article published in *Nature*, addressing kinship practices in the early Neolithic Tomb of Hazleton North, UK (Fowler *et al*. 2022), drew on terms such as patrilineality and patrilocality to explain patterns seen in the data. Thus, it is through the terminology of kinship as developed in social-cultural anthropology that its study is back on the archaeological agenda for debate, in a refashioned form from the writing of Gordon Childe (1950) or debates in the 1980s and ’90s (Sherratt 1981; Gimbutas 1991). As this recent resurgence is driven largely by aDNA research in archaeology, and hence is prioritizing biological kin, it has mostly been conducted by specialists not specifically trained in socio-cultural aspects of kinship studies: in consequence, there is a danger that many of the lessons learned about the social construction of kinship will fall away (see Carsten 2004; Cveček 2024; Godelier 2011; Goldfarb & Bamford 2024; Parkin [1997] 2003; Sahlins 2013). As **Scaffidi** (this issue) notes, genetic studies of Andean kinship have increased dramatically, along with the number of individuals analysed. However, interpretations often continue to reflect older anthropological approaches and rarely address how kinship is ‘produced, maintained, and contested’, as emphasized in current socio-cultural anthropology.

## Inferring biological relatedness through ancient DNA

Biological relationships ground much of the analysis and interpretation in archaeogenetics. These relationships vary in scope and timescales. At the broadest level, inferring genetic groupings and population structure using methods such as principal component analysis and ADMIXTURE/STRUCTURE involves assessing shared genetic features or, in other words, genetic similarities or relationships. In the past decade, developments in both data generation and analysis methods have spurred more aDNA studies and, concurrently, expanded the range of questions to which aDNA techniques can be applied. In particular, several archaeogenetics studies are starting to provide resolution at the regional and even site-specific scales (Ávila-Arcos *et al*. 2023; Racimo *et al*. 2020), which includes, among other insights, biological kinship. An array of computational methods has been developed to investigate genetic relatedness between individuals as well as parental relatedness over time, accounting for the low-quality and damaged nature of aDNA data (see review by Vai *et al*. 2020). **Amorim and Raff** (this issue) provide an overview of kinship-focused insights that archaeogenetic studies have provided.

The results have ranged from inferences of family units and elaborate pedigrees at a site to the identification of both close and more distant relatives across sites and generations, using a range of methods developed especially for low-quality aDNA data, e.g. READ, NgsRelate, lcMLkin, BREADR, KIN, hapROH and ancIBD (Alaçamlı *et al*. 2024; Korneliussen & Moltke 2015; Popli *et al*. 2023; Ringbauer *et al*.2024 2021; Rohrlach *et al*. 2023; Žegarac *et al*. 2021), and also see Vai *et al*. (2020) for a review of some of these methods. Moreover, the layering of other data forms such as archaeological context, radiocarbon dating, biological sex and isotope analysis have further helped in pedigree resolution (see Fowler *et al*. 2022; Rivollat *et al*. 2023; Schroeder *et al*. 2019; Yaka *et al*. 2021a,b; Yüncü *et al*. 2025). Most of these methods have been applied to ancient individuals from Europe and western Asia, among the regions with the densest aDNA data. This has led to capturing a lot of ancient European and western Asian traditions of genetic relatedness while missing many other forms of relationships that have occurred in the past. However, contributions of these methods to archaeological questions on past societal structure and the resolution that they afford are spectacular for the purpose they were developed for—inference of genetic relatedness, which may or may not equal kinship (see above).

## Testing old theories with new data: regional co-existence of different kinship systems

Early approaches to kinship, such as those of Morgan (1871), placed human kinship systems on a trajectory from primitive to civilized, proposing that matrilineal classificatory systems evolved into patrilineal classificatory systems, ultimately culminating in civilizations with ‘natural’ nuclear families characterized by descriptive kinship terminology (such as those followed in many westernized cultures).^11^ Following the archaeogenetic revolution, we stand at a point where some of those old kinship theories can be tested through well-dated archaeological contexts. Unlike the earlier claims that Neolithic societies were matrilineal (Gimbutas 1991; Sherratt 1981), recent archaeogenetic evidence from Neolithic contexts in Europe and western Asia, if taken at face value, has been interpreted as pointing towards patrilineal descent (Fowler *et al*. 2022; Rivollat *et al*. 2023; Schroeder *et al*. 2019) as well as potentially matrilineal lineages (Yüncü *et al*. 2025). Moreover, matrilocality appears to be the rule in Iron Age Britain (Cassidy 2025), long after the Bronze Age communities in Lech Valley are thought to have followed a patrilineal descent system and patrilocality (Mittnik *et al*. 2019).^12^ Hence, a unilineal development of kinship from matrilineal to patrilineal alongside increasing complexity does not appear to be the case in European prehistory. Instead, a co-existence of communities that followed different descent and/or residence patterns better explains this emerging pattern. For the first time, a combination of ancient DNA, stable isotopes and archaeological contexts can be used to challenge assumptions made about the evolution and change of past kinship systems.

## Kinship trouble and ancient DNA: genetic relatedness is not a universal way of becoming kin

While there is great potential in archaeogenetic research on kinship, archaeogenetics also contributes to kinship trouble when these biological relationships are ascribed kinship terms that derive from the socio-cultural anthropological literature, which, as discussed previously in this article, are not always reflected in entirety or accurately in genetic data. Socio-cultural anthropology and archaeology, to varying degrees, report on the diversity and fluidity in kinship structures across time and space. Hence, imposing a singular kinship structure, viewed from the Eskimo-type descriptive kinship systems as used in English (see above), defaults to a present-day westernized perspective in archaeogenetics studies. Inferred genetic relationships conflate socio-cultural and self-ascribed identities with biological identities, leading to widening gaps and tensions between the fields when they should be equal parties at the table (see Amorim & Raff, this issue; **Moots, Tsosie & Somel**, this issue).

There is no one single response to this development of, and increase in, aDNA research involving archaeological collections. Some archaeologists have readily embraced the methods as revealing social structures wholesale through comparing biological relatedness with material correlates, particularly in the funerary realm (e.g. Kristiansen 2022). This work is often carried out in combination with isotopic analysis of mobility, placing primacy on biological parent–offspring relationships (e.g. Haak *et al*. 2008) and descent and inheritance (e.g. Knipper *et al*. 2015), as prioritized forms of social belonging, often uncritically taking up the distance in biological relationships as the distance experienced socially (see Frieman & Schuster, this issue). Another disciplinary response has its origins in responding to this body of aDNA work, overwhelmingly driven by the critique of biological relationships as the universal foundation for kinship (Booth *et al*. 2021; Brück 2021; Cveček 2024; Frieman & Brück 2021), and how aDNA data and archaeological context should be interpreted in light of each other (Carlin *et al*. 2025; Fowler *et al*. 2022; Smyth *et al*. 2025). What emerges from this literature is several keys areas of kinship trouble faced today; the epistemological and ontological approaches to biological and social relations, including non-human kin (Crellin & Harris 2020); how to research, integrate and interpret archaeological and material proxies for kinship, such as houses or funerary practices (see Cveček 2023; 2025a; Ensor 2013; 2021; Ensor *et al*. 2017; Frieman & Schuster, this issue; Schroeder *et al*. 2019; Souvatzi 2017; Yaka *et al*. 2021a,b; Yüncü *et al*. 2025); and the extent to which fragmentary parts can be brought together to tell continent-wide narratives of human kinship (Mittnik & Bentley, this issue).

## *Why* does kinship trouble matter?

In the introduction to her and Donna Haraway’s short intervention into population growth and human-related climate change, *Making Kin not Population*, Adele Clarke (2018) calls for us to figure social belonging on different scales to counter the ways in which the global community faces harm.^13^ She argues that certain forms of belonging and relating, which prioritize biology as fixed and kinship as immutable, maintain these harms. Thus, on a fundamental level, kinship trouble matters to how we perceive the ways we are related to each other and our imagined distance from other members of our communities and beyond them. For this special issue, this is the ethical basis for the archaeological, anthropological and genetic approaches to kinship in the past: as we chart the global variability through time, we propose new possibilities in the present for relating to those around us and underscore that these possibilities, for the most part, can only be successfully implemented through inter-disciplinary and community-engaged methods. In more specific ways for each discipline, kinship trouble intersects with issues of boundaries: who leads research and who we choose to listen to in proposing interpretations, what kinds of research are prioritized for funding and publication, and which audiences we present our findings to and in what form.

Addressing past kinships along somewhat independent trajectories means that the potential for aDNA and archaeology to contribute back to anthropologies of kinship is vastly under-explored. In socio-cultural anthropology, kinship is understood as experienced in highly variable ways. Based on ethnographic documentation, different substances (e.g. blood, bone, sperm) could be crucial for local conceptions of kinship, which could change over a lifetime^14^ (Carsten 2000; Franklin 2013). Consuming food from the same hearth or within the same house may have been an important kinning practice, understood as physically making people kin through consumption of shared substance coming to make the body (Carsten 1997; Carsten & Hugh-Jones 1995). Adoption and fostering can happen at frequencies unthought of among western communities (Alber 2013). These notions are often (though not always) developed as a fictional anthropological present. Therefore, addressing kinship practices through case studies across the time depth offered by archaeology may not only further the anthropological understanding that there is no universally applicable pattern of kinship, but also bring attention to the changeable nature of kinship.

For example, **Bamford** (this issue) demonstrates that posthumous conception by means of using frozen sperm is not an ‘invention’ of the West but was in a different way practised and documented also among the Nuer in Sudan. At the same time, **Cveček and Gingrich** (this issue) refer to the Nuer practice of posthumous marriage, the so-called ‘ghost marriage’, to explain the possibility of levirate^15^ unions among the Avar as inferred through the recent ancient DNA study (Gnecchi-Ruscone *et al*. 2024). This special thematic issue goes beyond the current state-of-the-art by initiating a dialogue on kinship between scholars employing natural sciences and social sciences techniques to reach their conclusions about past kinship patterns as well as by reading individual contributions together to untangle the kinship trouble and promote interdisciplinary dialogue. This matters, in particular, to overcome the two cultures divide^16^ (Snow 1959) by developing a common language of some of the key anthropological kinship concepts (see above) in archaeogenetics, archaeology and socio-cultural anthropology to promote balanced, nuanced, contextualized and ethical interpretations of ancient kinship practices. In particular, ethical approaches that integrate input from the contributing disciplines and relevant non-academic and community partners (see Supernant *et al*. 2020) are necessary to minimize inaccuracies and insensitivities in the interpretations and reconstructions of the past that can, in turn, also have detrimental socio-political consequences for Descendant and Indigenous communities.

**Figure 2.**
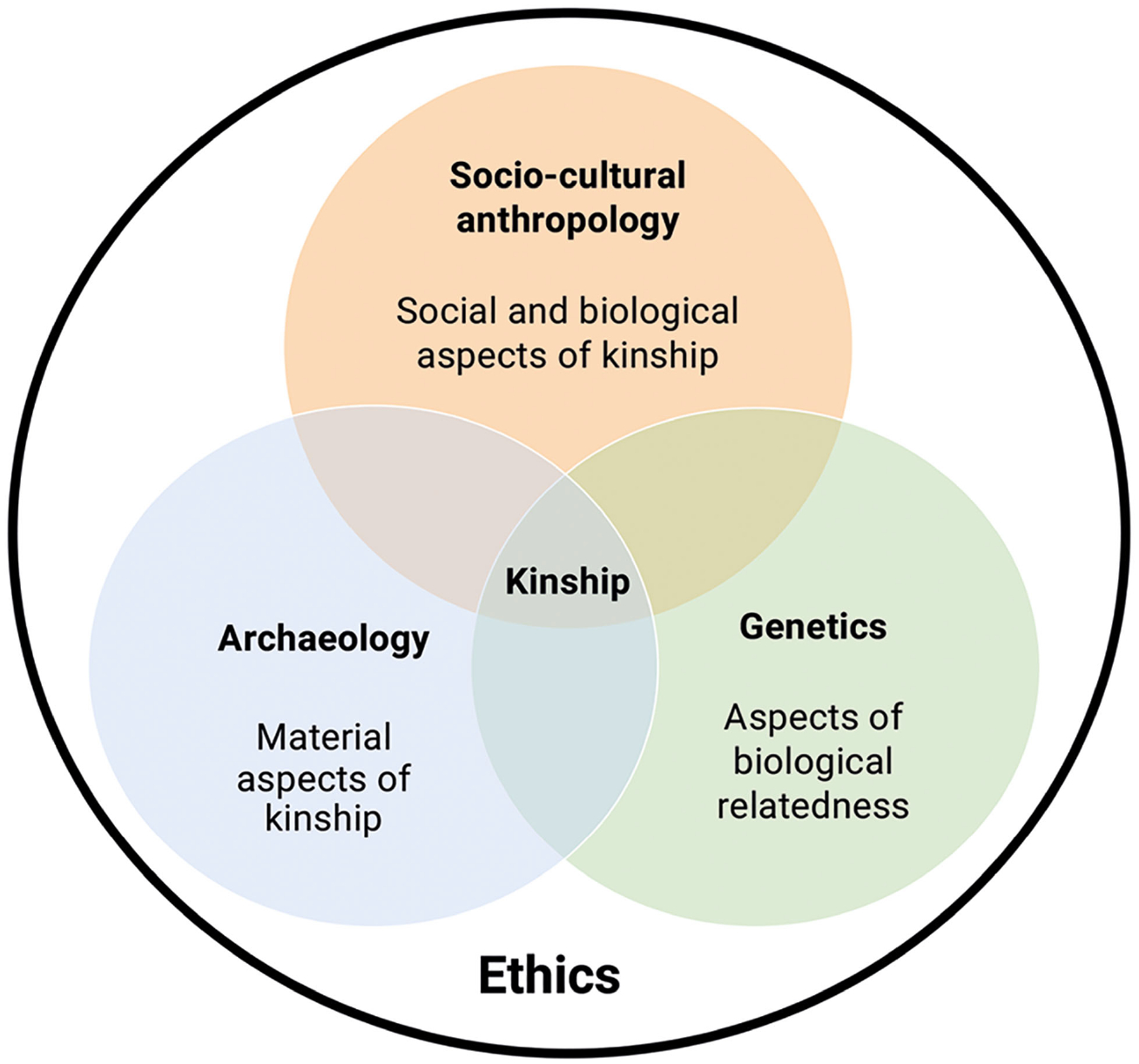
Ethical approaches underpin all steps of knowledge production within the ‘Kinship trouble’ framework.

Relevant to this special issue, ethical concerns have in fact been raised on kinship-related research, primarily in the North American context, that failed to consult and engage appropriately with stakeholder communities and, in some cases, prioritized DNA/biological kinship as the basis for repatriation (see Claw *et al*. 2017; Cortez *et al*. 2021; Fleskes *et al*. 2022; Moots *et al*., this issue). Kinship studies rooted in interdisciplinary and community-engaged (where relevant) investigations offer more holistic perspectives than each discipline alone and can hence promote more ethical research practices that enrich the scientific process and inferences while minimizing harms to communities (e.g. Kowal *et al*. 2023; Moots *et al*., this issue). Hence, ethical approaches underpin all steps of knowledge production within the kinship trouble framework, as depicted in Figure 2.

## *How* could kinship trouble be addressed?

**Figure 3.**
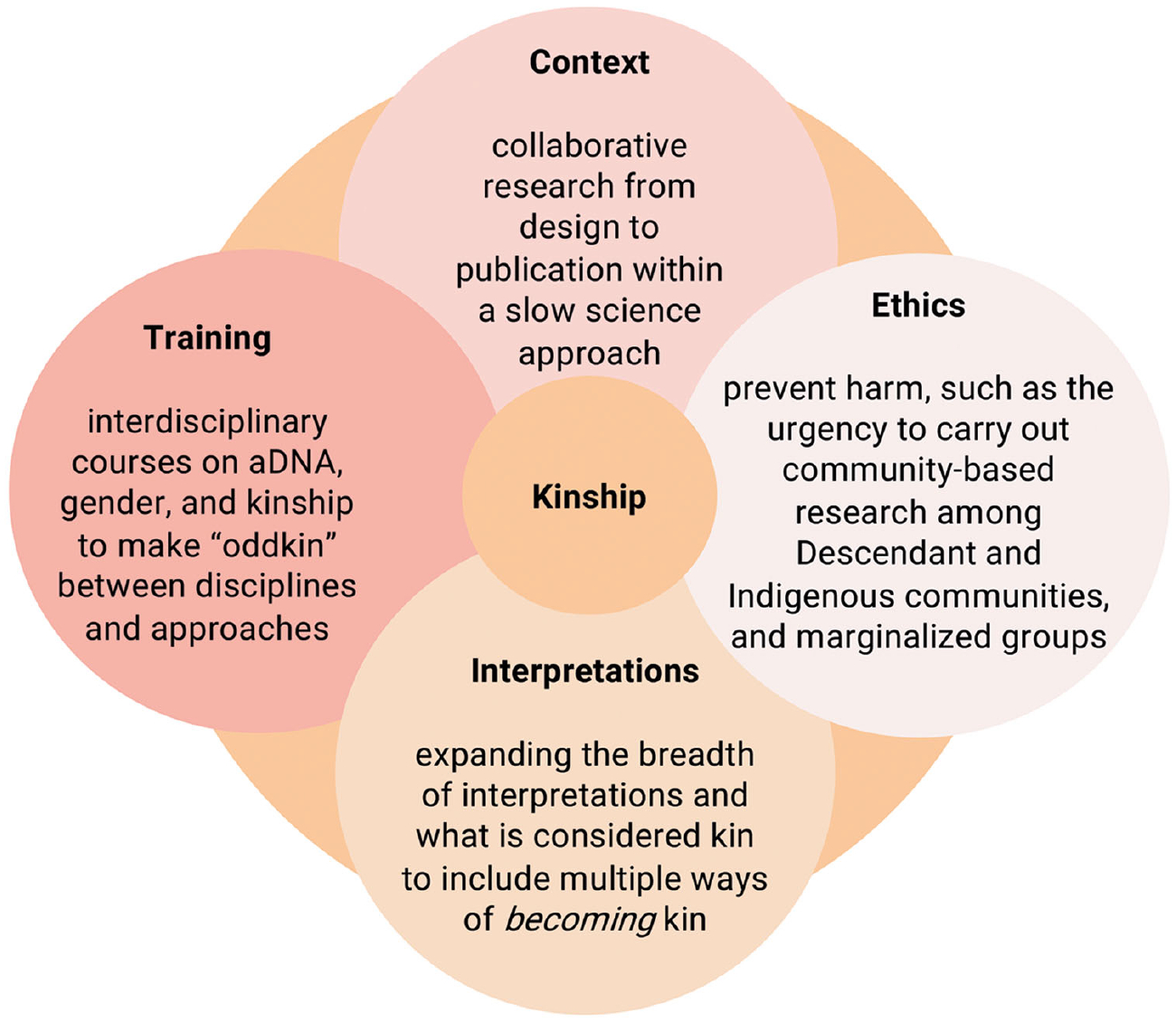
Four ways forward to address the kinship trouble through ethics, training, contexts and interpretations.

The current use of ancient DNA to trace biological relatedness to explain human behaviour and social organization in the past tells only one side of the story. Archaeogenetics alone cannot capture the complex intertwining of biological and social ties but results in kinship trouble instead. To resolve it, staying with the trouble (cf. Haraway 2016) is necessary. This requires making ‘oddkin’,^17^ meaning seeking out unexpected collaborations and combinations across disciplines, similar to the approach proposed by Donna Haraway (2016) for addressing the challenges of the Anthropocene. Staying with kinship trouble would also enable us to include the long-standing tradition of using specific kinship-related terms and concepts in socio-cultural anthropology in more nuanced, informed and precise ways, as already previously proposed (Cveček 2024; 2025a). For this shift to happen, however, much-needed renewed collaboration between archaeologists and socio-cultural anthropologists (Parkinson 2017) must also extend to archaeogenetics on the topic of kinship (Cveček 2024; 2025b; Cveček *et al*. 2025). To meet these challenges head-on, we identify four important avenues forward, which concern *training*, *context*, *interpretations* and, above all, *ethics*, as depicted in Figure 3.

As noted above, kinship as a topic of analysis remains an important topic of anthropological syllabi outside the US; modules on the topic are rare in archaeological degrees, and, depending on the institution, without much exposure to the socio-cultural underpinnings in genetics curricula. We propose advancing *training* in this field of research, through interdisciplinary courses (noting these are easier to organize in some institutional settings than others), perhaps starting with curated reading lists, would provide the foundation for integrating different topics such as aDNA, gender and kinship, in which there is an exploratory space for both student and teacher to sit with the ‘oddkin’ in different disciplines and to develop curiosity about other disciplinary histories and approaches. By research *context*, we move from training to the places and forms in which research is funded and carried out. We advocate for research projects which are founded as collaborative initiatives from the designing of research questions, and selection of samples, to the sources of funding and places of publication. This may mean slowing the research process down, in line with the call for slow science approaches (see Conkey 2024), something which may be uncomfortable for genetics research, so that kinships can be made between researchers across disciplines. It also demands that archaeology, which in some times and places has rather narrowly focused on kinship as revealed in genetic data, draws back out and takes seriously the proposals from anthropology (and some of its own members, e.g. Carlin *et al*. 2025; Ensor 2013; 2021; Frieman 2023; Meller *et al*. 2023; Rebay-Salisbury *et al*. 2021; Souvatzi 2017; Smyth *et al*. 2025; Whittle 2024) that kin relations are material and materialized in a broader range of contexts than the biology of the body, such as settlements and the funerary sphere.

It is to be hoped that such expansion of the contexts of collaboration would lead to balanced and informed *interpretations*. Slowing down the research production line from inception to publication would, we propose, leave space for more balanced and cautious interpretations of ancient kinship, as well as curiosity as to how we can expand our interpretations. We can expand our conceptions of what is considered kin; there is no reason that our aDNA results should not sit alongside discussion of how past peoples drew animals, materials and other concepts, such as gender, into their formations of kinship. It is here that we draw on the fourth dimension of kinship trouble, namely *ethics*. It is paramount, where relevant, for the interdisciplinarity framework to be inclusive of Descendant, Indigenous and marginalized communities throughout the research process as previously proposed by several authors (e.g. Bardill *et al*. 2018; Claw *et al*. 2017; Fleskes *et al*. 2022; Kowal *et al*. 2023; Moots *et al*., this issue; Wagner *et al*. 2020). Promoting collaborative and interdisciplinary research could lead to the eradication of existing asymmetries in ancient DNA research practices between countries in the Global North and Global South (see Argüelles *et al*. 2022) and the discontinuing of often unethical practices of sampling and analysing DNA from Ancestors globally (Källén 2025; Källén *et al*. 2024). We advocate strongly for community-based aDNA research, but also for awareness of how kinship studies have developed through time (see Figure 3). We have seen how ignorance of the histories of migration studies in archaeology has allowed aDNA results to be drawn into damaging ‘fortress of Europe’ narratives with real impacts for contemporary politics (Frieman & Hofmann 2019). Not one of us, working in the worlds of human relationships whether biological or social, can escape the political dimensions of research, and this trouble needs to be met head-on, and without fear.

## Kinship trouble: so what?

Kinship has long provided anthropology and archaeology with a powerful analytical framework, offering insights into social organization, alliance and identity. Yet, the very centrality of kinship also creates limitations from an investigative standpoint: theories that reduce it to rigid categories such as descent or alliance overlook the lived fluidity of relatedness, while archaeological and genetic data capture only fragments of how kinship was practised in the past. Ethical constraints further arise when Western notions of family are imposed in other global contexts or when Descendant community perspectives are disregarded. Thus, while kinship opens valuable avenues for understanding human societies, it also carries conceptual, methodological and ethical limits that require critical reflection and interdisciplinary dialogue.

### So, what, then, about kinship trouble?

Beyond identifying methodological challenges, the notion of kinship trouble foregrounds why interdisciplinary collaboration matters. Addressing ‘kinship trouble’ requires more than the application of anthropological theories and terminology to archaeological contexts by archaeologists or aDNA specialists; it calls for genuine dialogue across disciplines in multiple ways. For example, archaeology’s deep theorization of material worlds provides avenues to embodied forms of kin and relatedness, which can sit alongside and beyond what is expressed in language. Interdisciplinary collaboration is essential not only to refine archaeological interpretations of kinship in the deep past, but also to demonstrate the continuing relevance of kinship studies for socio-cultural anthropology and generate a new synthesis across disciplines (see Whiteley 2018). Archaeology provides unique long-term datasets that make it possible to revisit, revise or even refute early anthropological theories of social change, which, prior to the development of aDNA techniques, were impossible to test empirically. At the same time, the ethnographic and cross-cultural insights of socio-cultural anthropology illuminate the diversity and variability of kinship practices documented from the late nineteenth century until today on a global scale. Crucially, this dialogue must also include the perspectives on kinship of Descendant communities (Jacob & Sabzalian 2022; Tallbear 2018), whose ethical claims and lived experiences are central to interpreting the past responsibly. Bringing these perspectives together strengthens archaeological models while also offering anthropology new comparative horizons, expanding its temporal depth, and grounding its theories in the social, material, and biological traces—and contemporary responsibilities—of millennia (see Figure 1).

The *so what* of kinship trouble, therefore, lies in its capacity to reshape how kinship is studied across disciplines, to expand the temporal and comparative horizons of anthropology, and to anchor archaeological interpretations in dialogues that are both scientifically rigorous and socially accountable.

## Conclusion

Becoming kin is a human universal. However, how and with whom humans become kin may vary profoundly. Kin ties may be created by birth, shared blood, sperm, or bone, but also through adoption, co-residence, commensality, marriage, adoption, milk kinship, shared human experience, or close relations between humans, animals and plants. Therefore, kinship is much more than what meets the archaeogenetic eye. This discrepancy between the social and the genetic ties, however, poses kinship trouble, a term that captures the difficulties in reconstructing ancient kin relations as well as an attempt to resolve them through interdisciplinary collaboration and ethically adequate approaches.

We argue for the critical urgency of understanding kinship again not only as a biological but also as a social phenomenon for the study of societies through archaeogenetic, archaeological and anthropological approaches. Instead of letting genetic relatedness be cut out of wider everyday, socio-political relations and contexts, an important next step in kinship analysis in the more recent or deep past is to acknowledge a multidimensional framework in which close genetic relatedness is seen as possibly overlapping with—but not necessarily equal to—kinship.

As we see it, this can be achieved through profound changes in *training*, *context*, *interpretations* and *ethics*, that promote 1) interdisciplinary courses on aDNA, gender, and kinship to make ‘oddkin’ between disciplines and approaches; 2) collaborative research from design to publication within a slow science approach; 3) expanding the breadth of interpretations and what is considered kin to include multiple ways of *becoming* kin; and 4) following ethical approaches that prevent harm and understand the urgency to carry out community-based research among Descendant and Indigenous communities as well as marginalized groups. By integrating these perspectives and approaches, we can begin to resolve the kinship trouble and develop a more holistic, responsible and intellectually rigorous approach to the past.

## Footnotes

1 See subsequent sections.

2 Following critiques by Schneider (1984), Weston (1991) and Carsten (2000; 2004), we avoid the use of ‘fictive kin’ as a category, recognizing instead that all kinship is culturally constructed and grounded in practices of relatedness. Hence, kinship cannot be reduced to a binary between biological and hence ‘real kinship’ *versus* non-biological ties as ‘fictive kinship’.

3 See Judith Butler’s lecture here: https://www.youtube.com/watch?v=ixwrw0PMC8I

4 When we say kinship is about ‘becoming’ rather than ‘being’, we are emphasizing that relationships are often not just fixed by biology, but go beyond merely sharing DNA or being someone’s mother, father, cousin, etc. Instead, kinship is dynamic and often created and maintained through social, emotional, or cultural processes. It is about what people *do* to establish and nurture or break those connections, rather than just who they are genetically related to (see Carsten 2004).

5 For an important anthropological intervention on residence and house plans, see Hamberger (2018).

6 Exogamy refers to the marriage rule that prescribes a person to marry outside their kin group (see also **Cveček & Gingrich**, this issue).

7 Affinal ties are relations through marriage.

8 In socio-cultural anthropology, ‘an emic model is one which explains the ideology or behaviour of members of a culture according to indigenous definitions. An etic model is one which is based on criteria from outside a particular culture. Etic models are held to be universal; emic models are culture-specific.’ (Barnard & Spencer 2002, 275–7).

9 The term ‘new kinship studies’ refers to a body of literature in feminist anthropology that addresses gender, personhood, queer, gay and lesbian kinship (Andrikopoulos & Spronk 2023; Gzesh 2022; Weston 1991) and new reproductive technologies following Strathern (1992).

10 Structural functionalism was a theoretical school in Great Britain originally formulated to move away from evolutionism and diffusion. Structural functionalism, founded by Radcliffe-Brown (1941) and advanced by Evans-Pritchard (1940), focused on social structures over biological needs, viewing societies as organisms where local customs rather than biology maintain stability. Structural-functionalist approaches emphasized systematic, globally comparative approaches that nevertheless recognize the relevance of local specificities and exceptions as well as regional variations, without insisting on any *a priori* universals (while not excluding them; e.g. incest taboo among humans being one of those recognized universals).

11 However, even in the urban United States the ideal of the nuclear family does not apply universally. In *All Our Kin*, Carol B. Stack (1974) describes life in The Flats, where families facing systemic oppression, including racism and economic marginalization, developed flexible household boundaries, fluid co-residence arrangements and extensive community networks that extended well beyond nuclear family units.

12 See Mittnik & Bentley, this issue, for a complete list of sites where kinship analyses have been conducted.

13 Their volume covers legacies of slavery and violent othering of race, western hegemonies, and ecological and technological changes.

14 This may also be important for thinking about how geneticists design sampling when working with communities that may consider some tissues or body substances as being informative of different forms of kinship.

15 Levirate, also known as fraternal polyandry, refers to marriage arrangements in which a girl/bride/woman marries her husband’s brother after the death of her husband (see Parkin [1997] 2003, 43).

16 Charles Percey Snow’s (1959) ‘two cultures’ divide highlighted the gap between the sciences and the humanities, arguing that their lack of communication and the divide between the two hinder solving global issues. He advocated bridging this divide to foster interdisciplinary collaboration and scientific advancement.

17 Donna Haraway defines making ‘oddkin’ when ‘we require each other in unexpected collaborations and combinations, in hot compost piles. We become-with each other or not at all … in our separate kinds of expertise and experience, we know both too much and too little, and so we succumb to despair or to hope, and neither is a sensible attitude.’ (Haraway 2016, 4).
